# Expansion of CD57^+^ CD8 T cells in common variable immunodeficiency with hepatopathy and CMV infection

**DOI:** 10.3389/fimmu.2025.1577934

**Published:** 2025-05-27

**Authors:** Patrick Bez, Enrico Santangeli, Sigune Goldacker, Ulrich Salzer, Klaus Warnatz

**Affiliations:** ^1^ Division of Immunodeficiency, Department of Rheumatology and Clinical Immunology, Medical Center -University of Freiburg, Faculty of Medicine, University of Freiburg, Freiburg, Germany; ^2^ Center for Chronic Immunodeficiency, Medical Center - University of Freiburg, Faculty of Medicine, University of Freiburg, Freiburg, Germany; ^3^ Rare Diseases Referral Center, Internal Medicine I, Ca’ Foncello Hospital, Treviso, Italy; ^4^ Department of Pediatrics, Azienda Socio Sanitaria Territoriale (ASST) Spedali Civili of Brescia, Department of Clinical and Experimental Sciencies, University of Brescia, Brescia, Italy

**Keywords:** common variable immunodeficiency (CVID), immune phenotype, diagnosis, CD8 T cells, CD57, CMV infection, hepatopathy, splenomegaly

## Abstract

**Background:**

Common variable immunodeficiency (CVID) is associated with an altered immune homeostasis affecting many T-cell subpopulations, including an increased proportion of CD57^+^ CD8 T lymphocytes. This expansion has been associated with the clinical manifestation of granuloma/lymphadenopathy and a positive CMV status. The aim of the study is to describe the prevalence of an expansion of CD57^+^ CD8 T cells in CVID patients and determine its diagnostic value.

**Methods:**

This is a monocentric retrospective study including 131 patients with a median follow-up of 9 years. The inclusion criteria are a diagnosis of CVID according to European Society for Immunodeficiencies (ESID) criteria and at least two independent assessments of CD57^+^ CD8 T cells. Patients on immunosuppressive therapy were excluded.

**Results:**

The expansion of CD57^+^ CD8 T cells was part of the previously described immune alteration, including altered CD4/CD8 ratio and a decrease in naïve CD4 T cells. The loss of significant association with increasing age might corroborate the suggestion of premature immunosenescence in CVID. Significantly higher values of CD57^+^ CD8 T cells were seen in patients with a complicated clinical phenotype, and especially associated with the presence of splenomegaly, status post-splenectomy, and hepatic disease. Additionally, patients with a history of CMV infection presented with elevated CD57^+^ CD8 T cell values. When comparing the potential diagnostic value of expanded CD57^+^ CD8 T cells compared to alterations in other T-cell subsets in relation to specific complications, we could not identify a single complication in CVID patients for which absolute or relative CD57^+^ CD8 T cell counts were superior to more commonly used T-cell populations, except for CMV infection.

**Conclusion:**

This is the largest study on the prevalence and diagnostic relevance of the expansion of CD57^+^ CD8 T cells in CVID. Most CD57^+^ CD8 T cells are part of the CD45RA^+^ terminal effector subset. While we could not detect an added value of the diagnostic evaluation of CD57^+^ CD8 T cells at this time, further investigation in circulation and tissue might enhance our understanding of the pathogenesis of hepatic disease and thereby gain novel diagnostic value in the future.

## 1 Introduction

Common variable immunodeficiency (CVID) is the most common symptomatic primary immunodeficiency disorder. It is characterized by reduced serum immunoglobulins, impaired vaccine response and reduced class-switched memory B cells (1). Besides the increased risk of mainly bacterial infections, up to 50% of CVID patients present with immunodysregulatory manifestations such as autoimmune cytopenia, lymphoproliferation, non-infectious enteropathy, hepatopathy and interstitial lung disease (2). Therefore, CVID was classified into two clinical phenotypes: infection-only CVID (CVIDio) and those who suffer also from immunodysregulatory complications (CVIDc) (3).

Multiple alterations of the homeostasis of the adaptive immune system have been identified in CVID patients. While the reduction of class-switched memory B cells is found in nearly all CVID patients, others are of diagnostic value for identifying patients with the manifestation of non-infectious complications. These include the expansion of CD21^low^ B cells according to the EUROclass classification (4) and signs of impaired T-cell homeostasis (5–7), such as the reduction of naïve CD4 T cells (8, 9).

Among CD8 T cells, an increased proportion of total CD8 T cells—and in particular effector memory CD8 T cells (T_EM_) and terminal effector cells re-expressing CD45RA (T_EMRA_)—has been reported in CVIDc patients with autoimmune cytopenia (9, 10) and local expansion and alteration of circulating CD8^+^ T cells in enteropathy (11). More recent reports used deep phenotyping and found an increase of exhausted-like CD8 T cells in CVID patients with ILD and lymphoproliferation and of activated CD38^+^HLA-DR^+^ CD8 T cells in patients with enteropathy (12). The increase in the proportion of CD57^+^ CD8 T lymphocytes compared to healthy donors in CVID patients (13) has previously been associated with granuloma/lymphadenopathy and CMV-positive status (14, 15). Among CD8 T lymphocytes, CD57 (known formerly as Human Natural Killer 1 or LEU7) is mainly observed on CD8 T_EM_ and T_EMRA_ (16), and has been considered a marker of senescence (17, 18). Different levels of expression of CD57 may be related to telomere length and INF-y production (16). The aim of the study is to describe the expansion of CD57^+^ CD8 T cells in a cohort of CVID patients followed up at the Centre for Chronic Immunodeficiency (CCI) in Freiburg over time and explore its diagnostic value in association with clinical and other laboratory parameters.

## 2 Methods

The inclusion criteria of this retrospective study are (I) a diagnosis of CVID according to European Society for Immunodeficiencies (ESID) criteria (19), (II) the availability of immunophenotyping, including an evaluation of the percentage of CD57^+^ CD8 T cells at least two time points at least 5 months apart, and (III) absence of immunosuppressive therapy at the time of phenotyping except for low-dose steroids (i.e., ≤ 7.5 mg/day). The study was approved by the Ethics Committee of the University of Freiburg (EK No. FR251/13 and FR354/19) and complies with the Helsinki Declaration of 1964. All patients have signed an informed consent form. The clinical and genetic data were collected from the clinical records in a pseudonymized way from 04/2023 to 07/2023. An informatics query was performed in July 2023 to obtain data on extensive T- and B-cell immune phenotypes, blood tests, EBV-DNA, and CMV-DNA (01/2004-12/2022).

### 2.1 Definitions

Splenomegaly was defined as a longitudinal diameter >13 cm on ultrasound. Enteropathy was defined as chronic diarrhea and considered severe when marked weight loss (>5% of body weight) due to enteropathy occurred. Hepatopathy was diagnosed when liver enzyme elevation occurred for more than 6 weeks and/or a diagnosis of hepatopathy was found in the clinical records. Portal hypertension was considered in the presence of evidence of esophageal varices and/or ascites. Expansion of CD57^+^ CD8 T cells was defined according to the reference values of the diagnostic laboratory of the CCI Freiburg as a percentage of CD57^+^ CD8 T cells >32% in those under 45 years and >53% in those above 45 years of age.

### 2.2 Flow cytometric analysis

The immunological laboratory at the University Medical Center Freiburg assesses circulating lymphocytes by flow cytometry using a basic panel for the enumeration of T, B, and NK cells, T-cell specific panel for the enumeration of CD3+CD4+, CD3+ CD8+, activated HLA-DR+ CD4 T cells and CD8 T cells, CD45RA+ naive and CD45RO+ memory CD4 T cells; TCR gamma-delta+ and alpha-beta+ T cells, CD127neg CD25+ regulatory CD4 T cells, and different populations of memory CD8 T cells, including CD57+ CD8+ T cells. The B-cell panel allows for the classification of CVID patients according to the Euroclass trial4 and additionally the detection of IgG+ and IgA+ switched memory B cells (smB). In this study, we query the following data: lymphocyte, total T cells, total NK, total CD4 T cells, total CD8 T cells, and total CD19 B cells in counts/µl; HLA-DR+ CD4 T cells, HLA-DR+ CD8 T cells; naïve CD4 T cells, late effector CD28-CD27- CD8 T cells, and early effector CD28-CD27+ CD8 T cells; CD57+ CD8 T cells; CD21lo B cells, IgD-CD27+ smB. The following antibodies were used for the analysis of CD57+ CD8+ T cells: anti-CD57 FITC (clone NC1, Beckman-Coulter), anti-CD28 PE (clone L293, Becton Dickinson), anti-CD8 PerCP (clone SK1, Biolegend), anti-CD27 PE-Cy7 (clone M-T271, Becton Dickinson), anti-CD45RA APC (clone MEM-56, Exbio), and anti-CD3 BV421 (clone UCHT1, Biolegend). Cells were analyzed on a Navios flow cytometer (Beckman-Coulter), and data were analyzed with the help of Kaluza 2.1 Softwar (Beckman-Coulter). **Supplementary Figure 1** shows the gating strategy for CD57+ CD8 T cells.

### 2.3 Statistics

R 4.3.3 software (R Core Team. R: A Language and Environment for Statistical Computing. R Foundation for Statistical Computing, Vienna, Austria. https://www.R-project.org. 2023; package Tidyverse) was used to perform descriptive analysis and inferential analysis: the Mann-Whitney U-test and Kruskal-Wallis test with *post-hoc* Dunn’s test (dunn.test package) were used to compare continuous non-parametric unpaired variables; the Wilcoxon test was used for non-parametric paired variables; the Fisher’s exact test and McNemar’s test were used for unpaired and paired observation, respectively, for categorical variables. The Shapiro-Wilk test for normality and the Spearman rank test were used to evaluate correlation. Statistical Package for the Social Sciences (SPSS) was used to perform data modeling. The tests were considered significant when the p-value was smaller than 0.05.

## 3 Results

### 3.1 General characteristics

According to the inclusion criteria, we selected 131 CVID patients out of 663 patients attending the CCI outpatient clinic at the University Medical Center Freiburg between 2000 and 2023. The available immune phenotyping and interval between the phenotypes are reported in **Supplementary Table 1**.

The median age of the patients at the last follow-up is 46 years (Interquartile range, IQR, 22-41), and the median duration of follow-up is 9 years (IQR 6-23). A total of 75 (57%) patients were female, and 88 (67.5%) patients presented with a complicated phenotype (CVIDc). The general characteristics and clinical complications of the cohort are summarized in **Table 1**. Genetic testing was available in 108 (82.4%) patients and yielded a conclusive diagnostic result in 31 patients (29%). The most frequently reported monogenic diagnoses were CTLA-4 haploinsufficiency and NFKB1 haploinsufficiency and carriers of mutations in *TNFRSF13B*. The genetic results are summarized in **Supplementary Table 2**.

**Table 1:** Patient characteristics at last follow-up.

Characteristics	TOTAL (N=131)
Sex (Female) (%)	75 (57.3%)
CVIDc (%)	88 (67.2%)
Dead (%)	6 (4.6%)
Genetic testing (%)	108 (82.4%)
Age at onset* (Median, IQR, range)	22 (16-35)
Age at diagnosis (Median, IQR, range)	33 (22-41)
Age at last FU (Median, IQR, range)	46 (36-58.5)
Years of FU (Median, IQR, range)	9 (6-23)
Splenomegaly*	82 (63.5%)
Splenectomy	8 (6.1%)
Generalized lymphadenopathy	31 (23.7%)
Granuloma (confirmed by biopsy)	17 (13%)
Interstitial lung disease	29 (22.1%)
Autoimmune cytopenia	33 (25.2%)
AIHA	16 (12.2%)
AITP	27 (20.6%)
Chronic diarrhea	37 (28.2%)
Severe enteropathy	13 (9.9%)
Hepatopathy	24 (18.3%)
Portal hypertension	10 (7.6%)
Hematologic neoplasia	10 (7.6%)
Cancer	11 (8.4%)

The categorical variables are expressed as absoulte and relative (%) frequency. The continous variable are expressed as median and interquartile range (IQR). CVIDc, complicated CVID; AIHA, autoimmune cytopenia; AITP, autoimmune thrombocytopenia. *Data available for 129 patients.

At the time of phenotyping, the patients were not receiving any immunosuppressive therapy (besides low-dose steroids). In total, 94 patients (72%) did not receive immunosuppressive therapies 5 years before and between the first two samplings; 17 patients (13%) had received systemic immune suppressive therapies in the 5 years preceding the first time point; 16 patients (12%) received systemic immune suppressive therapies between the first two time points; an additional 4 patients (3.1%) received systemic therapies before and between the sampling.

### 3.2 Comparison of the phenotypes over time

Considering that three and four time points of phenotyping were available only in 25% and 7.5% of patients, respectively, we performed the analysis by comparing only the first two immune phenotypes. The two phenotypes were performed with a median of 4 years apart (IQR 2.8-5.9). Several statistical differences were noted between the immune phenotypes, albeit the effect size is small or negligible. We found a slight decrease in absolute lymphocyte counts, affecting CD4 T cells, CD8 T cells, and B cells but not NK cells. Furthermore, we observed a slight increase in the relative but not absolute CD57^+^ CD8 T cell counts, a slight decrease in the percentages of HLA-DR^+^ CD8 T cells, early effector T cells, and naïve CD4 T cells of their respective parent population (**Supplementary Table 3**). The B-cell phenotype, according to EUROClass, remained fairly stable over this period of time, showing no relevant differences between both time points in regard to the main subclasses. The analysis of the patients with a confirmed genetic diagnosis found no statistical difference in the relative or absolute counts of CD57^+^ CD8 T cells when compared to patients without detectable mutations (**Supplementary Table 2** and **Supplementary Figure 2**).

### 3.3 Clinical characteristics at time of phenotyping and at last follow-up

**Figure 1:**
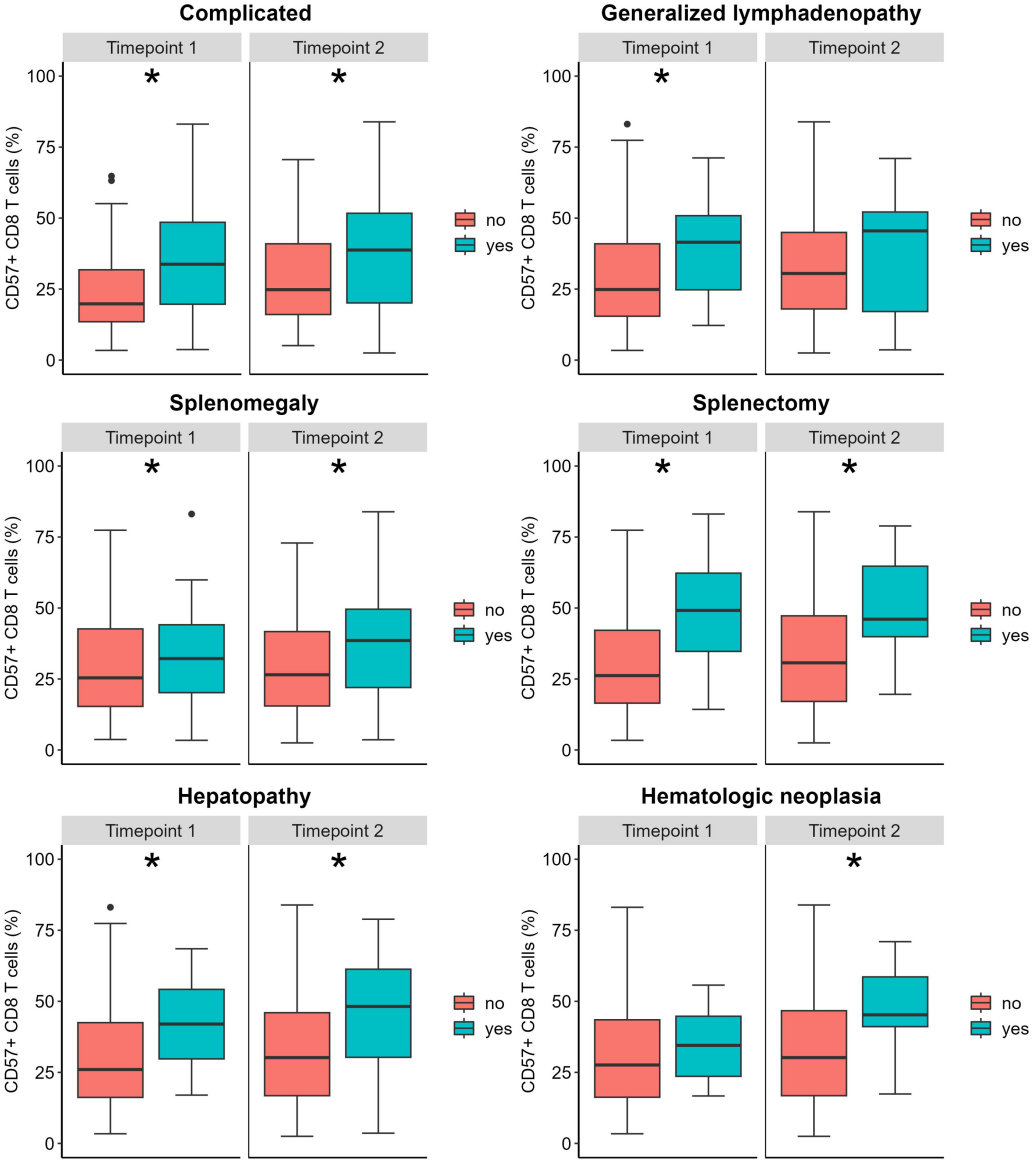
Relative CD57^+^ CD8 T count according to clinical complications. The box plot represents the median and the interquartile range; the vertical line represents the range of the 5th and 95th percentiles; the dots represent outliers (values above or below the 5th and 95th percentiles, respectively). The Mann-Whitney U test was used to compare distributions at each time point (see **Table 2**). *Indicates a p <0.05.

We found a weak positive correlation between age and percentage of CD57^+^ CD8 T cells only at the second timepoint of phenotyping (timepoint 1 (t1): R=0.145, p=0.096; timepoint 2 (t2): R=0.241, p=0.006, respectively). CVIDc presented with higher levels of CD57^+^ CD8 T cells than CVIDio (t1: p=0.002, t2: p =0.007), but no differences between sexes were observed. We evaluated the difference in CD57^+^ CD8 T cell percentage in relation to the different complications present at the time of immune phenotyping (**Figure 1** and **Supplementary Figure 3**). We could identify higher values in patients with splenomegaly, the status post splenectomy, and hepatic disease at both time points and for lymphadenopathy for the first time point and for lymphomas for the second time point. None of the patients developed a CD57^+^ large granular T cell lymphoma. We did not find statistical differences for patients suffering from granuloma, GLILD, enteropathy, autoimmune cytopenia, or history of solid malignancy (**Table 2**). Next, we evaluated the predictive value of an increased percentage of CD57^+^ CD8 T cells at the first or second time point in patients who would only subsequently develop hepatopathy up to July 2023, as the prevalence of this complication increased from 10 (7.6%) at the first to 18 (14%) at the second timepoint and up to 24 (18%) in July 2023. There was no detectable difference between the group of patients with or without new onset of hepatopathy (Kruskal-Wallis test t1: p=0.279 and t2: p=0.406). It was not possible to perform a similar analysis for autoimmune cytopenia, splenomegaly, and status post-splenectomy because these complications appeared early in the course of the disease, and almost no patients developed these complications after the first time point of evaluation.

**Table 2:** Comparison of the percentage of CD57^+^ CD8 T cells by clinical presentation.

Characteristics	Timepoint 1			Time point 2		
	No	Yes	p	No	Yes	p
Sex (Female)	29.7 (17.5-49.3)	24.9 (16.2-42)	0.310	33.85 (21.1-46.9)	26.5 (17.1-48.2)	0.388
**CVID Complicated phenotype**	**19.8** **(13.5-31.8)**	**33.75** **(19.7-48.5)**	**0.002**	**24.8** **(16-41)**	**38.75** **(20.1-51.7)**	**0.007**
**Generalized lymphadenopathy**	**25.5** **(16.2-41.2)**	**41.65** **(30-52.4)**	**0.012**	31.1 (18.6-45.7)	43.45 (16.7-51.7)	0.442
**Splenomegaly**	**18.4** **(12.2-33.1)**	**33.4** **(20.1-46.4)**	**0.002**	**21.2** **(15-41.2)**	**39.8** **(22.4-51)**	**0.002**
**Splenectomy**	**26.2** **(16.5-42.2)**	**49.15** **(34.7-62.3)**	**0.039**	**30.7** **(17.1-47.2)**	**46.05** **(39.9-64.7)**	**0.037**
Granuloma	26.1 (16.4-42.7)	39.3 (17-50.1)	0.287	32.8 (17.8-46.6)	29.8 (17-49)	0.843
GLILD	26.1 (16.3-43.2)	33.2 (18.6-46)	0.395	30.9 (18.4-46.2)	35.3 (16.4-51.7)	0.526
AIC	28.6 (16.2-43.8)	24.6 (17-41.3)	0.914	33 (17.7-47.6)	29.7 (17.2-47.6)	0.956
AITP	28.6 (16.3-43.8)	23.8 (17-41.3)	0.977	32.9 (17.8-48)	30.3 (16.4-46.1)	0.909
AIHA	28.6 (16.2-43.5)	22.8 (19.3-42.9)	0.902	32.8 (17.7-47.1)	29.1 (17.4-57.2)	0.820
Enteropathy	26.2 (16.1-42.5)	31.85 (20.1-49.7)	0.232	30.7 (16.8-46.1)	35.45 (19.1-52.1)	0.544
**Hepatopathy**	**26** **(16.2-42.5)**	**42** **(29.8-54.2)**	**0.041**	**30.2** **(16.8-46)**	**48.2** **(30.3-61.3)**	**0.008**
**Hematologic malignancy**	27.6 (16.2-43.5)	34.5 (23.6-44.8)	0.387	**30.2** **(16.8-46.7)**	**45.25** **(41.1-58.6)**	**0.023**
Cancer	27.6 (16.3-44.1)	29.05 (19.9-31.2)	0.813	31.1 (17.6-47.8)	36.25 (25.4-48.5)	0.574

The Mann-Whitney U test was used to compare the distribution of CD57+ CD8 T cells in patients with or without a clinical characteristic at the time of phenotyping. All values reflect median and interquartile range in brackets. GLILD, granulomatous/lymphocytic interstitial lung disease; AIC, Autoimmune Cytopenia; AITP, Autoimmune Thrombocytopenia, AIHA, Autoimmune Hemolytic Anemia.

Significant variables are depicted in bold.

### 3.4 Correlations with other laboratory features

We evaluated the correlations between CD57^+^ CD8 T-cell percentage and other laboratory features. The GGT values were the only laboratory feature with a weak to moderate correlation to CD57^+^ CD8 T-cell percentage at both time points (t1: R=0.288, p = 0.001; t2: R=0.388, p < 0.001). The sIL2R/WBC (white blood cell counts) ratio correlated weakly with this T-cell subset (t1: R=0.223, p=0.034; t2: R=0.231, p=0.025), whereas sIL2R merely missed significance at the second time point (t1: R=0.279, p=0.008; t2: R=0.202, p=0.050), while both relative and absolute neutrophil counts showed a significant reversed correlation at this time point (**Supplementary Table 4**). Similarly, GGT elevation was the only laboratory alteration associated with significantly higher levels of CD57^+^ CD8 T cells (t1: p<0.001; t2: p<0.001) (**Supplementary Table 5**). No such associations were found when we considered absolute values (data not shown).

### 3.5 Associations with other immune cell subsets

We found a strong correlation between CD57^+^ CD8 T cells with late effector T cells (t1: R=0.831, p<0.001; t2: R=0.847, p<0.001), moderate positive correlations between the percentage of activated HLA-DR^+^ CD4 and CD8 T cells, and weak correlations between absolute and relative total CD8 T cells. On the other hand, we observed moderate negative correlations with the CD4/CD8 ratio and naïve CD45RA^+^ CD4 T cells and weak negative correlations with the percentage of total CD4 T cells. We found no correlations of this population with either absolute or relative B and NK cells. We found a weak negative correlation with switched memory B cells (t1: R=-0.191, p=0.033; t2: R=-0.205, p=0.024), but no correlation with CD21^low^ B cells (**Table 3**). Considering Euroclass B cell classification (4), we stratified the patients into five Euroclass groups (namely B-, smB+CD21norm, smB+CD21lo, smBCD21norm, and smBCD21lo). The levels of CD57+CD8T cells were different among groups (Kruskal-Wallis test <0.001 at both time points), in particular, the *post-hoc* Dunn’s test showed that smB+CD21norm showed lower levels than the other four groups (see **Supplementary Tables 6**, **7** and **Supplementary Figure 4**).

**Table 3:** Correlation of the percentage of CD57^+^ CD8 T cells with other immune cell parameters. .

Cell type	First time point		Second Time point	
	p	R	p	R
WBC	0.468	0.064	0.276	-0.095
Lymphocyte % (of total WBC)	0.029	0.189	0.015	0.211
Lymphocyte abs (cell/mm^3^)	0.071	0.158	0.326	0.087
T cell % (of total lymphocytes)	0.222	0.107	0.144	0.128
T cell abs (cell/mm^3^)	0.027	0.192	0.169	0.121
CD4 T cell % (of total T cells)	**0.007**	**-0.233**	**<0.001**	**-0.307**
CD4 T cells abs (cell/mm^3^)	0.650	0.040	0.583	-0.048
CD8 T cell % (of total T cells)	**<0.001**	**0.329**	**<0.001**	**0.374**
CD8 T cell abs (cell/mm^3^)	**<0.001**	**0.293**	**0.002**	**0.264**
Ratio CD4/CD8	**<0.001**	**-0.315**	**<0.001**	**-0.401**
HLA-DR^+^ CD4 T cell % (of total CD4 T cells)	**<0.001**	**0.455**	**<0.001**	**0.554**
Naïve CD45RA^+^ CD4 T cell % (of total CD4 T cells)	**<0.001**	**-0.387**	**<0.001**	**-0.338**
HLA-DR^+^ CD8 T cell % (of total CD8 T cells)	**<0.001**	**0.524**	**<0.001**	**0.579**
Early effector (CD28^-^CD27^+^) % (of total CD8 T cells)	0.493	0.059	0.109	-0.140
**Late effector (CD28^-^CD27** ^-^ **) % (of total CD8 T cells)**	**<0.001**	**0.831**	**<0.001**	**0.847**
NK cells % of total lymphoctyes	0.170	-0.120	0.224	-0.107
NK abs (cell/mm^3^)	0.788	0.024	0.935	0.007
CD19^+^ B cell % of total lymphocytes	0.278	-0.096	0.384	-0.083
CD19^+^ B cells abs (cell/mm^3^)	0.765	-0.026	0.673	-0.037
smB cells % of total B cells*	0.033	-0.191	0.024	-0.205
CD21^low^ cells % of total B cells*	0.058	0.171	0.178	0.123

Spearman correlation test comparing the relationship between populations. Abs, absolute values; %, relative values; WBC, white blood cells. *In patients with a B+ phenotype (B cell > 1%).

Significant variables are depicted in bold.

### 3.6 Association with chronic viral infections

Some CVID patients are confronted with chronic virus infections such as CMV, EBV, or norovirus. Thus, we were interested in whether the expansion of CD57^+^ CD8 T cells was associated with either of these viruses. Copy numbers of plasma CMV-DNA were determined in 91 patients (69%) and suggested a history of CMV infection in 15 (16% of our cohort), five of whom presented with a clinically relevant complication (one colitis, one pneumonia, one with liver involvement and cytopenia, and two with systemic infections). Patients with a history of positive CMV-DNA presented with higher percentages and absolute values of CD57^+^ CD8 T cells at both time points (**Table 4**). No differences were found in relative and absolute numbers of CD57^+^ CD8 T cells in patients with or without a history of EBV or chronic norovirus infection (**Table 4**).

**Table 4:** Comparison of relative and absolute numbers of CD57^+^ CD8 T cells by history of chronic viral infection. .

	type	N (%)	- / +	Timepoint 1			Timepoint 2		
				No	Yes	p	No	Yes	p
CD57^+^ CD8 T cell %	**CMV**	**91** **(69%)**	**76/15**	**28.1 (16.8-44.9)**	**41.8 (37.6-48.6)**	**0.023**	**30.5 (16.4-46.9)**	**51.7 (43.2-57.9)**	**0.001**
	EBV	73 (56%)	61/12	33.9 (19.7-50.1)	35.2 (27.7-48.5)	0.738	34.7 (21.7-49.2)	48.2 (41-55.1)	0.113
	Norovirus	53 (40%)	41/12	26 (18.1-42.9)	26.9 (14.8-46.1)	0.758	39 (23.9-52.2)	30.4 (19.1-49.8)	0.531
**CD57^+^ CD8 T cell abs**	**CMV**	**91** **(69%)**	**76/15**	**81 (36-168)**	**196 (134-442)**	**0.007**	**77 (31-192)**	**175 (110-382)**	**0.007**
	EBV	73 (56%)	61/12	90 (39-255)	141 (42-159)	0.76	87.8 (44-256)	119 (71-178)	0.760
	Norovirus	53 (40%)	41/12	132 (47-222)	97 (35-185)	0.456	97 (56-185)	169 (29-196)	0.456

The Mann-Whitney U test was used to test for differences between groups. Virus positivity was assessed by PCR for the respective viruses in plasma (CMV, EBV) or stool (norovirus). Parameters with significant differences are presented in bold. All values reflect median and interquartile range in brackets. abs, absolute value (cell/mm^3^); %, relative value; N, number; -/+ patients with negative PCR/at least one positive PCR result.

### 3.7 Diagnostic value of CD57^+^ CD8 T-cell expansion

Stratifying patients into patients with normal or elevated percentage of CD57^+^ CD8 T cells according to the criteria mentioned in the Methods section demonstrated an increased presence of splenomegaly at both time points (t1: p=0.042, t2: p=0.035) and status post-splenectomy only at first time point (p=0.045), whereas the association with CMV history and hematologic neoplasia was present only at the second timepoint (p=0.003 and p=0.035, respectively) in patients with elevated CD57^+^ CD8 T cells (**Supplementary Table 8**). A male predominance at time point 1 was not seen at the second time point. None of the incidences of the other clinical presentations were increased in patients with elevated CD57^+^ CD8 T cells, including hepatopathy. To assess whether a classification of CVID patients according to the relative values of CD57^+^ CD8 T cells compared to other subpopulations could be clinically relevant, we investigated the values of CD57^+^ CD8 T cells and other T-cell subsets in relation to the manifestation of hepatopathy, splenomegaly, splenectomy, and CMV infection. Regarding hepatopathy and splenomegaly, we observed increased CD57^+^ CD8 T cells, activated HLADR^+^ CD8 T cells, and HLA-DR^+^ CD4 T cells and reduced naïve CD45RA^+^ CD4 T cells. Moreover, patients with splenomegaly tended to present with lower levels of lymphocytes, especially CD8 T cells (see **Supplementary Tables 9**, **10** and **Supplementary Figures 5**, **6**). Concerning splenectomy, we observed increased levels of total T cells, including both CD4 T cells and CD8 T cells, and absolute values of CD57^+^ CD8 T cells. In this regard, we observed very high absolute values of CD57^+^ CD8 T cells in the first 2 years after splenectomy (**Supplementary Table 11** and **Supplementary Figure 7**). For CMV, we observed higher values of both absolute and relative CD57^+^ CD8 T cell counts and percentage of activated HLA-DR^+^ CD8 T cells and late effector CD8 T cells (**Supplementary Table 12** and **Supplementary Figure 8**). In summary, absolute or relative CD57^+^ CD8 T cell counts did not add clinically relevant diagnostic information to the more commonly used absolute CD4 and CD8 counts and percentage of naïve CD4 cells, except for CMV infection.

## 4 Discussion

An increased proportion of CD57^+^ CD8 T cells has been described in various immune-mediated disorders, including inborn errors of immunity, such as Activated Pi3K delta syndrome (APDS) (20) and Del22q11 syndrome (21), in autoimmune disorders such as rheumatoid arthritis (22), and in infectious diseases such as HIV (23) and CMV (24). Also, in CVID, this population has been previously found to be increased (14). However, data on the characterization of this population over time and in large CVID cohorts are missing in order to evaluate its diagnostic value. To the best of our knowledge, this is the largest series evaluating the expansion of CD57^+^ CD8 T cells over time in CVID patients in regard to their clinical and immune phenotype.

The strong positive correlation of relative counts of CD57^+^ CD8 T cells with the percentage of late effector memory (CD28^-^CD27^-^) CD8 T cells suggests most of this population is a terminally differentiated CD8 T-cell population, as previously described by Kuntz et al. (14). The expansion of CD57^+^CD8 T cells is part of a broader alteration of the immune phenotype in CVID patients. As previously reported in CVID, we observed a positive correlation of CD57^+^ CD8 T cells with HLA-DR^+^ T cells, suggesting an activated phenotype (25, 26), and a negative correlation with CD4/CD8 ratio (27). The negative correlation with naïve CD45RA^+^ CD4 T cells is consistent with the association of both alterations with a complicated CVID phenotype (8, 28). The lower proportion of CD57^+^ CD8 T cells in patients belonging to the B+smB+21norm group compared to all other groups according to Euroclass (4) further supports the possible link of this population with a complicated phenotype. Previously, our group did not identify any correlation with B-cell phenotyping, possibly due to the lower number of patients analyzed (34 patients) (14).

We found neither a difference in CD57^+^ CD8 T cells between genders nor a consistent association of this population with age in our cohort, which has been observed in the general population (29). This may reflect a premature immunosenescence in a relevant proportion of CVID patients (25). While certain monogenetic CVID disorders might be more prone to lead to an expansion of CD57^+^ CD8 T cells, this was not specific nor diagnostically helpful, as we found strongly overlapping values between genetically undefined and monogenetic CVID patients included in this cohort.

Considering the clinical features of our patients, we found a higher proportion of CD57^+^ CD8 T cells in CVIDc compared to CVIDio patients. This discrimination has also been seen for the EUROClass classification (4), however, the profile of the associated manifestations with the respective immune phenotype was distinct. In contrast to the previously reported associated increase in CD57^+^ CD8 T-cell percentage with granuloma (14, 30) lymphadenopathy (14), and GLILD, (31, 32) in the present study, we found significantly higher proportions of this subset only in patients with splenomegaly, status post-splenectomy, and hepatic disease. Consistent with these results, we found correlations with GGT values, sIL2R/WBC ratio, and a borderline correlation with sIL2R as a marker of T-cell activation (33). The discrepancy of our results may be due to a difference in size and selection criteria of the cohorts and the exclusion of patients on immunosuppressive therapy. About splenectomy, we observed higher absolute and relative levels of CD57^+^ CD8 T cells in patients with splenectomy compared to those without. Viallard et al. have recently reported an increase in CD3^+^ lymphocytes, especially CD8 T cells, and CD19^+^ B cells following splenectomy (34). They did not analyse the marker CD57 in their T-cell panel. Our data may suggest that the majority of the circulating CD8 T cells following splenectomy are CD57^+^. Unfortunately, lacking sufficient CD57^+^ CD8 T-cell data prior to splenectomy means that more definitive conclusions are not possible.

Among the viral infections analysed, our data suggest an association of elevated absolute and relative CD57^+^ CD8 T-cell counts with a history of CMV infection, as previously described in CVID and in the general population (14, 29). No relevant associations for EBV and norovirus infection were identified.

Then, analyzing the CD57^+^ CD8 T-cell expansion according to the laboratory threshold, we could confirm only the associations with splenomegaly. Still, we must consider that these thresholds were set in healthy donors and were not studied specifically for CVID to identify different complications.

Finally, we wanted to know whether there is a diagnostic added value in the evaluation of CD57^+^ CD8 T cells in general and compared to currently suggested other cell populations. Currently, the reduction of CD4 T cells and the naïve CD4 T-cell percentage (8) are used in clinical practice as potential markers of complicated phenotype and of organ-specific complications, such as GLILD and hepatopathy. Therefore, we sought to determine whether CD57^+^ CD8 T-cell assessment has a superior diagnostic utility in the diagnosis of patients with hepatopathy, splenectomy, splenomegaly, and CMV infection. While the percentages of CD57^+^ CD8 T cells were significantly increased in patients with liver disease, as a single marker, it was not sufficient to distinguish patients with and without this manifestation and was inferior to the more established investigation of reduced naïve CD4 T cells. Unfortunately, the percentage of CD57^+^ CD8 T cells did not allow for predicting the development in patients who develop hepatopathy during the observation period. CD57^+^ CD8 T cells were superior in our cohort to distinguish patients with status post-splenectomy, which might be of interest in understanding the role of large spleens in regard to homing of different lymphocyte populations but is of no diagnostic value. This is also true for the association with splenomegaly, as this is readily identified by ultrasound and also associated with multiple other already established cell markers in CVID diagnostics, such as lymphopenia (34), low naïve CD4 T cells (35), and expansion of CD21^low^ B cells (4). The association with a history of CMV infection is interesting; it has also been observed in healthy donors (14, 29). The diagnostic value of this association remains not completely understood until we have a better understanding of the effect of CMV infection on the clinical course of CVID and therefore does not seem to justify a routine evaluation of CD57 expression on T cells for diagnostic purposes. Unfortunately, the diagnostic potential for granulomatous manifestations of CVID, which are often difficult to diagnose, was also insufficient to justify its routine inclusion.

The strength of this study is that it is the largest series evaluating the diagnostic value of CD57^+^ CD8 T cells in CVID. Its limitations include the retrospective character, so that the intervals between phenotypes were not standardized. The exclusion of patients with immunosuppression at the time of phenotyping (n=28) to ensure that the data were not altered by this treatment may have reduced the prevalence of certain immunodysregulatory complications in the cohort. The positive detection of the copy number of CMV, EBV, or norovirus may have underestimated the prevalence of the respective viral infection in our cohort (especially for asymptomatic patients), but this was the only reliable way, as serological testing is not possible due to the ongoing immunoglobulin replacement therapy. Finally, due to insufficient data beyond the third time point and the non-normal distribution of the samples, we were unable to apply generalized linear models for repeated measures among the lymphocyte subsets at different time points to identify trends. Therefore, we applied a conservative approach with non-parametric data analysis and considered a result to be relevant only when consistent at both time points.

In summary, CD57^+^ CD8 T cells in CVID seem to resemble a late effector subset and to be associated with a chronic activation of the immune system. As such, this population is associated with a complicated phenotype and specific clinical complications, in particular hepatopathy, splenomegaly, and splenectomy. Furthermore, we could confirm higher levels of this population in patients with a history of CMV infections, supporting the notion of a potential role of chronic activation and premature immunosenescence. However, overall, we conclude that the anticipated minor diagnostic value of CD57^+^ CD8 T cells does not justify their addition to diagnostic panels in the current clinical practice. Yet, in regard to the increasingly recognized role of hepatopathy in the prognosis of the disease, it will be of high research interest to combine the evaluation of CD57^+^ CD8 T cells in tissue biopsies and circulation to improve our pathogenetic understanding of this complication, which may justify its reevaluation for diagnostic purposes.

## Data Availability

The data are not readily available. Specific request need to be sent to the corresponding author.
