# Supplementary material for: Expansion of CD57+ CD8 T cells in common variable immunodeficiency with hepatopathy and CMV infection
Source: Front Immunol. 2025 May 27;16:1577934. doi: 10.3389/fimmu.2025.1577934 (PMC12149213; doi:10.3389/fimmu.2025.1577934)
Supplement: Supplementary file 1 [file Table1.pdf]

EXPANSION OF CD57+ CD8 T CELLS IN COMMON VARIABLE IMMUNODEFICIENCY (CVID) WITH HEPATOPATHY AND CMV INFECTION

Patrick Bez†[1,2,3], Enrico Santangeli†[1,4], Sigune Goldacher[1,2], Ulrich Salzer[1,2], Klaus Warnatz[1,2*]

1. Division of Immunodeficiency, Department of Rheumatology and Clinical Immunology, Medical Center - University of Freiburg, Faculty of Medicine, University of Freiburg, Freiburg, Germany;
2. Center for Chronic Immunodeficiency, Medical Center - University of Freiburg, Faculty of Medicine, University of Freiburg, Germany
3. Rare Diseases Referral Center, Internal Medicine I, Ca' Foncello Hospital, AULSS2 Marca Trevigiana, Treviso
4. Department of Pediatrics, ASST Spedali Civili of Brescia, Department of Clinical and Experimental Sciencies, University of Brescia, Brescia, Italy

† These authors contributed equally to this work and share first authorship.

SUPPLEMENTARY TABLES

Supplementary table 1. Number of available immune phenotypes

	N (%)	Interval in years (Median, IQR)
1st phenotype (range)	131 (100)	NA
2nd phenotype (range)	131 (100)	4.0 (2.8-5.9)
3rd phenotype	31 (25)	2.0 (0.84-3.3)
4th phenotype	7 (5.3)	1.4 (0.73-3.2)

N refers to the number of patients with immunophenotyping including the evaluation of CD57$^+$ CD8 T cells. interval in years refer to the median number of years to the respective previous analysis. Abbreviations: NA not applicable. IQR Interquartile range

Supplementary Table 2. Comparison of relative and absolute CD57 + CD8 T cell counts by genetic result

**Time point**	**CD57+ CD8 T cell**	***CTLA-4*** 6 (5.5%)	***NFKB1*** 10 (9.3%)	***TNFRSF13B*** 9 (8.3%)	**Negative** 77 (71%)	**p**
1	Relative value	18.8 (16.4-26)	21.1 (9.4-31.7)	31.8 (23.6-38.7)	28.6 (17-44.9)	0.150
	Absolute values	33.3 (19.4-70.7)	82.5 (47-142)	83.5 (48.3-263)	89.2 (43.4-241)	0.234
2	Relative value	25 (17.8-33.5)	26.3 (20.1-48.6)	32.8 (17.7-43.8)	35.3 (16.8-49.7)	0.356
	Absolute values	56 (10.2-94.8)	132 (82.9-186)	80.2 (49.9-308)	87.8 (49.4-257)	0.324

Genetic testing was available in 108/131 (82%) patients. Other genetic results not displayed in the tables comprise single mutations in *IKAROS, NFKB2, SOCS1,* and *FAS*. The Kruskal-Wallis test was used to test difference among the groups. The percentage of CD57+ CD8 T cells are expressed as median and interquarile range.

Supplementary Table 3 Comparison of immune phenotypes over time

	1st phenotype	2nd phenotype	p
**CD57$^+$ CD8 T cell %**	**27.7 (16.5-43.5)**	**32.8 (17.7-47.9)**	**<0.001**
CD57$^+$ T cell abs	87 (40-225)	95 (46-205)	0.255
WBC (cell/mm^3)	5818 (4682-7718)	5900 (4670-7353)	0.129
**Lymphocytes % (of total WBC)**	**22.9 (16.7-29.4)**	**21.8 (15.4-27.1)**	**0.021**
**Lymphocytes abs**	**1274 (926-1825)**	**1163 (794-1726)**	**<0.001**
**T cell % (of total lymphocytes)**	**80.1 (73.4-85.9)**	**78.4 (69.8-86.5)**	**0.003**
**T cell abs** (cell/mm^3)	**1023 (766-1434)**	**897 (597-1350)**	**<0.001**
CD4 T cell % (of total T cells)	44.4 (35.7-53.4)	44.2 (34.8-53.3)	0.317
**CD4 T cells abs** (cell/mm^3)	**584 (413-780)**	**522 (360-684)**	**<0.001**
CD8 T cell % (of total T cells)	28.8 (22-35.8)	28.3 (20.6-36.2)	0.406
**CD8 T cell abs** (cell/mm^3)	**380 (236-573)**	**308 (182-560)**	**0.003**
Ratio CD4/CD8	1.56 (1.07-2.3)	1.3 (0.9-2.2)	0.977
HLA-DR$^+$ CD4 T cell % (of total CD4 T cells)	11.3 (5.95-18.6)	9.9 (5.2-17.9)	0.266
**CD45RA$^+$ CD4 T cell % (of total CD4 T cells)**	**17.3 (8.8-33.0)**	**16.2 (7.5-29.6)**	**0.002**
**HLA-DR$^+$ CD8 T cell % (of total CD8 T cells)**	**21.6 (12.1-32.4)**	**18.9 (11.2-31.8)**	**0.022**
**Early effector (CD28$^-$CD27$^+$) CD8 T cell % (of total CD8 T cells)**	**9.7 (6.4-15.5)**	**8.6 (5.3-14.9)**	**0.019**
Late effector (CD28$^-$CD27$^-$) CD8 T cell % (of total CD8 T cells)	25 (10.9-51.5)	23.4 (11.0-52.5)	0.204
NK cells % (of total lymphoctyes)	94 (54-145)	102 (53-156)	0.910
**NK abs** (cell/mm^3)	**7.6 (4.8-10)**	**8.8 (5.2-13.9)**	**<0.001**
CD19$^+$ B cell % (of total lymphocytes)	11.9 (6.1-16.3)	10.6 (4.5-16.9)	0.123
**CD19$^+$ B cells abs** (cell/mm^3)	**134 (64-234)**	**128 (45-214)**	**<0.001**

The differences of lymphocyte populations were computed with Wilcoxon test. The lymphocytic populations are expressed as median and interquarile range. Parameters with significant differences are presented in bold. Abbreviations: WBC: white blood cells; %: percentage; abs: absolute value.

Supplementary Table 4. Correlation of the percentage of CD57+ CD8 T cells with laboratory parameters

	Time point 1			Time point 2		
	**N**	**R**	**p**	**N**	**R**	**p**
Hb (g/dl)	126	-0.027	0.764	130	-0.097	0.271
PLT (G/mm^3)	126	-0.018	0.840	130	-0.151	0.085
Total lymphocytes (cell/mm^3)	122	0.097	0.287	122	0.088	0.328
Neutrophils (% of total WBC)	121	-0.162	0.076	**127**	**-0.275**	**0.002**
Absolute Neutrophils	121	-0.092	0.313	**127**	**-0.235**	**0.007**
ALT (U/L)	118	0.180	0.052	**121**	**0.335**	**<0.001**
AST (U/L)	22	0.349	0.118	19	0.423	0.070
**GGT (U/L)**	**123**	**0.288**	**0.001**	**122**	**0.388**	**<0.001**
**sIL2R (U/L)**	**90**	**0.279**	**0.008**	94	0.202	0.050
**sIL2R/WBC**	**90**	**0.223**	**0.034**	**94**	**0.231**	**0.025**
Neopterin (nmol/l)	**27**	**0.434**	**0.023**	23	0.251	0.249

Laboratory parameters were tested on the same day of immune phenotyping at both time points. The correlation was computed with Spearman Rank test considering the non-parametric distribution of CD57+ CD8 T cells. Parameters with significant differences are presented in bold. Abbreviations: N= the number of data available; R=coefficient of Spearman correlation test; Hb: Hemoglobin; PLT: platelet, WBC: white blood cells; ALT: alanine transferase; AST: aspartate transferase; GGT: gamma-glutamyl transpeptidase; sIL2R: soluble interleukin receptor 2.

Supplementary Table 5. Relative CD57$^+$ CD8 T in patients stratified by specific laboratory abnormalities

	At 1st phenotype			At 2nd phenotype		
	**No**	**Yes**	**p**	**No**	**Yes**	**p**
Hb <100 g/L	26.1 (16.3-42.3)	49.5 (43.8-52.2)	0.063	27.7 (16.3-44.1)	33.6 (33.6-33.6)	0.064
PLT <100 G/ mm^3	26.9 (16.3-42.8)	36.1 (25.7-47.1)	0.345	27.6 (16.3-42.7)	38 (22.8-45.4)	0.092
Neutropenia (<1500 cell/ mm^3)	25.8 (16.2-41.6)	34.3 (20.1-42.9)	0.382	27.7 (16.6-44.1)	28.5 (21.1-39.2)	0.382
Lymphopenia (<1000 cell/mm^3)	26.9 (15.7-44.2)	27.3 (19.2-34)	0.775	28.1 (16.1-43.8)	25.8 (19-42.1)	0.782
ALT elevation (>50 U/L)	26.1 (16.6-41.8)	35.8 (17.1-55)	0.371	**27.7 (16.6-42.6)**	**40 (22.2-54.4)**	**0.049**
AST elevation (>50 U/L)	21.1 (16.3-31.8)	54.9 (48.4-59.3)	0.14	**19.7 (14.3-37.3)**	**40.1 (34.8-46.8)**	**0.032**
**GGT elevation (>60 U/L)**	**24.9 (15.2-41.1)**	**45.6 (27.9-56)**	**<0.001**	**24.7 (15.2-40.6)**	**42.4 (24.4-54)**	**0.001**
sIL2R elevation (>623 U/L)	**16.9 (11.7-30.1)**	**29.7 (17.1-42.9)**	**0.009**	26.4 (14.6-39.1)	28.1 (19.3-43.4)	0.203
Neopterin elevation (>10 nmol/l)	14.9 (14.9-14.9)	34.1 (24.6-42.4)	0.140	17.7 (17.7-17.7)	38.6 (21.2-43.8)	0.522

The comparison of the percentages of CD57$^+$ CD8 T cells between the respective groups is performed with the Mann-Whitney U test. Parameters with significant differences are presented in bold. The percentage of CD57+ CD8 T cells are expressed as median and interquarile range. Abbreviations: Hb haemoglobin; PLT: platelet; ALT: alanine transferase; AST: aspartate transferase; GGT: gamma-glutamyl transpeptidase; sIL2R: soluble interleukin receptor 2.

Supplementary table 6. Percentage of CD57+ CD8 T cells in patients stratified by Euroclass classification

	**Timepoint 1**			**Time point 2**		
	**N (%)**	**CD57+ CD8 T cells percentage**	**p**	**N (%)**	**CD57+ CD8 T cells percentage**	**p**
**B-**	7 (5.3%)	38 (27-44)	<0.001	11 (8.4%)	36 (17-47)	<0.001
**smB+CD21norm**	20 (15%)	17 (12-27)		20 (15%)	17 (14-22)	
**smB+CD21lo**	22 (17%)	24 (16-38)		25 (19%)	31 (22-44)	
**smB-CD21norm**	29 (22%)	29 (16-47)		31 (24%)	41 (25-52)	
**smB-CD21lo**	53 (40%)	34 (20-45)		44 (34%)	38 (22-50)	

Patients were stratified according to the Euroclass classification.[4] The percentage of CD57+ CD8 T cell percentage is expressed as median and interquartile range. The Kruskal-Wallis test was used for multiple test comparisons. For the single pairs comparisons, please refer to supplementary table 7. Abbreviations: N total number of patients; -/+ indicates number of subjects who did not or did belong to the specific Euroclass group. B+ indicates patients with a percentage of B cells >1% of total lymphocytes; smB- is considered when switched memory B cells were less or equal to 2% of total B cells; CD21lo is considered when $CD21^{low}CD19^{high}$ B cells were higher than 10% of total B cells.

Supplementary table 7. Difference of CD57+ CD8 T cells among groups according to EUROclass Classification.[4]

	**T1**	**T2**
**smB+CD21norm vs B-**	**0.016**	**0.019**
**smB+CD21norm vs smB-CD21lo**	**<0.001**	**<0.001**
**smB+CD21norm vs smB-CD21norm**	**0.01**	**<0.001**
**smB+CD21norm vs smB+CD21lo**	0.071	**0.003**
B- vs smB+CD21lo	0.130	0.441
B- vs smB-CD21norm	0.263	0.181
B- vs smB-CD21lo	0.429	0.217
smB-CD21lo vs smB+CD21lo	0.051	0.201
smB-CD21lo vs smB-CD21norm	0.199	0.406
smB-CD21norm vs smB+CD21lo	0.218	0.161

Patients were stratified according to the Euroclass classification.[4] The Kruskal Wallis test showed significant differences (please refer to supplementary table 6). The p values represent the results of Dunn's test to compare the difference in percentages of $CD57^{+}$ CD8 T cells between the respective groups at the respective timepoint. Parameters with significant differences are presented in bold. Abbreviations: B- indicates patients with a percentage of B cells <1% of total lymphocytes; smB- is considered when switched memory B cells were less or equal to 2% of total B cells; CD21lo is considered when $CD21^{low}CD19^{high}$ B cells were higher than 10% of total B cells.

Supplementary table 8. Comparison of the prevalence of an expanded CD57+ CD8 T cell population in patients by different epidemiological and clinical parameters

	Timepoint 1				Timepoint 2			
	**No (N=93)**	**Yes (N=38)**	**p**	**OR (95% C.I)**	**No (N=84)**	**Yes (N=47)**	**p**	**OR (95% C.I)**
Sex (Female)	**60 (65%)**	**15 (39%)**	**0.011**	**0.36 (0.15-0.84)**	52 (62%)	23 (49%)	0.200	0.59 (0.27-1.3)
Complicated phenotype	58 (62%)	30 (79%)	0.1	2.2 (0.88-6.3)	52 (62%)	36 (77%)	0.12	2 (0.85-5)
**Splenomegaly***	**54 (58%)**	**28 (78%)**	**0.042**	**2.5 (0.98-7.1)**	**47 (57%)**	**35 (76%)**	**0.035**	**2.4 (1.2-6.0)**
Splenectomy	**3 (3.2%)**	**5 (13%)**	**0.045**	**4.5 (0.82-30)**	3 (3.6%)	5 (11%)	0.130	3.2 (0.59-21)
Generalized Lymphadenopathy	12 (13%)	8 (21%)	0.290	1.8 (0.58-5.3)	17 (20%)	11 (23%)	0.660	1.2 (0.46-3.1)
Granuloma	13 (14%)	4 (11%)	0.777	0.73 (0.16-2.6)	11 (13%)	6 (13%)	1.000	0.97 (0.27-3.1)
GLILD	12 (13%)	7 (18%)	0.420	1.5 (0.46-4.7)	16 (19%)	13 (28%)	0.280	1.6 (0.64-4.1)
AIC	18 (19%)	7 (18%)	1	0.94 (0.3-2.7)	19 (23%)	11 (23%)	1	1 (0.4-2.6)
AITP	15 (16%)	6 (16%)	1	0.98 (0.28-3)	16 (19%)	9 (19%)	1	1 (0.36-2.7)
AIHA	9 (9.7%)	3 (7.9%)	1	0.8 (0.13-3.5)	8 (9.5%)	7 (15%)	0.400	1.7 (0.47-5.7)
Enteropathy	16 (17%)	6 (16%)	1	0.9 (0.26-2.7)	20 (24%)	14 (30%)	0.530	1.4 (0.56-3.2)
Hepatopathy	7 (7.5%)	3 (7.9%)	1	1.1 (0.17-4.9)	10 (12%)	8 (17%)	0.440	1.5 (0.48-4.7)
Hematologic neoplasia	5 (5.4%)	3 (7.9%)	0.690	1.5 (0.22-8.2)	**3 (3.6%)**	**7 (15%)**	**0.035**	**4.7 (1-29)**
Cancer	5 (5.4%)	1 (2.6%)	0.670	0.48 (0.01-4.5)	5 (6%)	3 (6.4%)	1	1.1 (0.16-5.8)
**CMV infection****	7 (11%)	8 (29%)	0.059	3.4 (0.93-12)	**4 (7.1%)**	**11 (31%)**	**0.003**	**5.8 (1.5-27)**

The p and odds ratio are calculated with Fisher's exact test. Parameters with significant associations are presented in bold. * data available in 129 patients. ** data available in 91 patients, 64 negative, 27 positive. Abbreviations: OR= odds ratio; GLILD= granulomatous and lymphocytic interstitial lung disease; AIC= autoimmune cytopenia; AITP= autoimmune thrombocytopenia; AIHA= autoimmune hemolytic anemia; C.I.=confidence interval.

Supplementary table 9. Comparison of different T cell populations in patients with or without hepatic disease

Population	Timepoint1<br>No	Yes	p	Timepoint 2<br>No	Yes	p
CD3+abs	1030 (783-1430)	736 (509-1470)	0.258	918 (621-1350)	790 (535-1090)	0.387
CD3+CD4+%	43.7 (35.7-52.2)	63.9 (35.8-76.6)	0.075	43 (35.1-53.2)	45 (33.7-60.1)	0.590
CD3+CD4+abs	584 (424-778)	512 (408-848)	0.921	527 (378-682)	461 (299-741)	0.833
**CD3+CD4+CD45RA+%**	**18.4 (9.8-33.2)**	**4.1 (2.73-11.4)**	**0.003**	**17.2 (9.4-30.2)**	**6 (3.38-17)**	**0.002**
**CD3+CD4+DR%**	**11 (5.5-17.5)**	**22.5 (18.3-26.4)**	**0.004**	**8.2 (4.5-15.5)**	**20.7 (14.3-28.6)**	**<0.001**
CD3+CD8+%	29 (22.4-34.9)	17.2 (12.9-38.9)	0.191	28.4 (21-35.6)	23.4 (15.4-36.4)	0.291
CD3+CD8+abs	386 (251-578)	140 (101-368)	0.055	315 (223-562)	232 (131-510)	0.213
**CD3+CD8+CD57+%**	**26 (16.2-42.5)**	**42 (29.8-54.2)**	**0.041**	**30.2 (16.8-46)**	**48.2 (30.3-61.3)**	**0.008**
CD3+CD8+CD57abs	88.1 (43.4-222)	50.9 (30.7-238)	0.588	95.1 (49.4-197)	97.1 (45.1-339)	0.657
**CD3+CD8+DR%**	**20.6 (11.8-30.7)**	**43.4 (38-51)**	**<0.001**	**17.4 (10.5-28.6)**	**33.2 (23.2-51.9)**	**0.003**
CD3+CD8+EE%	10 (6.9-14.9)	7.05 (5.93-19.3)	0.755	8.7 (5.3-14.9)	8.15 (5.05-13.8)	0.683
CD3+CD8+LE%	23.5 (10.5-51.4)	31.2 (21.2-66.3)	0.136	**21.7 (10.6-50.8)**	**35.5 (22.9-68.2)**	**0.027**

Mann-Whitney was used to compute the comparison. Parameters with significant associations are presented in bold. The lymphocytic populations are expressed as median and interquarile range. Abbreviations: % percentage; abs: absolute count; DR+: HLA-DR+; EE: early effector (CD28-CD27+); LE: late effector (CD28-CD27-).

Supplementary table 10. Comparison of different T cell populations in patients with or without splenomegaly

Population	Timepoint1<br>No	Yes	p	Timepoint 2<br>No	Yes	p
CD3+%	76.3 (72-82.3)	81.7 (75.1-87.4)	**0.006**	76.5 (70-81)	80.7 (68.6-88.4)	0.071
CD3+abs	1150 (876-1440)	958 (724-1320)	0.079	1090 (768-1390)	822 (558-1210)	**0.040**
CD3+CD4+%	42.6 (36.4-48.7)	46.6 (33.6-57.7)	0.331	39.4 (35.8-48.1)	44.8 (33-56.2)	0.260
CD3+CD4+abs	658 (492-780)	528 (392-764)	0.090	566 (400-713)	458 (307-616)	0.105
**CD3+CD4+CD45RA+%**	**29 (13.6-40.4)**	**15.4 (6.72-29.8)**	**0.001**	**25.6 (11.6-37.4)**	**13 (6.6-22.5)**	**<0.001**
**CD3+CD4+DR%**	**6.9 (3.8-12.8)**	**13 (8.43-22.8)**	**<0.001**	**5.4 (3.6-9.9)**	**13 (7-21.2)**	**<0.001**
CD3+CD8+%	29 (23.8-34.4)	28.4 (21.4-35.7)	0.529	30.6 (22.6-34.8)	25.2 (20.2-37.5)	0.188
CD3+CD8+abs	435 (274-572)	348 (190-562)	0.133	427 (240-583)	278 (148-537)	**0.036**
**CD3+CD8+CD57+%**	**18.4 (12.2-33.1)**	**33.4 (20.1-46.4)**	**0.001**	**21.2 (15-41.1)**	**39.8 (22.4-51)**	**0.001**
CD3+CD8+CD57abs	73.5 (40.7-137)	107 (39.3-236)	0.222	88 (50.7-150)	101 (41.6-228)	0.526
**CD3+CD8+DR%**	**15.9 (7.5-30.2)**	**25.6 (15.2-37.1)**	**0.002**	**13.4 (7.4-23.1)**	**24.3 (14.4-38)**	**<0.001**
CD3+CD8+EE%	8.2 (4.9-13.3)	11 (7.22-16.6)	0.015	7.7 (4.95-11.4)	10.4 (5.32-16)	0.089
CD3+CD8+LE%	16.6 (8-45.1)	29.1 (11.9-52.1)	0.060	17.1 (9.65-41.8)	29.9 (13-56.6)	0.051

Splenomegaly data are available in 129 patients. Mann-Whitney is used to compute the comparison. Parameters with significant associations are presented in bold. The lymphocytic populations are expressed as median and interquarile range. Abbreviations: % percentage; abs: absolute count; DR+: HLA-DR+; EE: early effector (CD28-CD27+); LE: late effector (CD28-CD27-).

.

Supplementary table 11. Comparison of different T cell populations in patients with or without splenectomy

Population	Timepoint1			Timepoint 2		
	**No**	**Yes**	**p**	**No**	**Yes**	**p**
**CD3+%**	**80.1 (73.2-85.7)**	**84.6 (77.9-91.1)**	**0.188**	**78.4 (70-85.5)**	**82 (68.8-92.2)**	**<0.001**
**CD3+abs**	**983 (745-1360)**	**2020 (1570-4130)**	**<0.001**	**866 (589-1240)**	**2500 (1770-4240)**	**<0.001**
CD3+CD4+%	44.6 (35.8-54.2)	41.7 (33.5-45.2)	0.451	44.2 (35-53.3)	43.9 (32.5-52.1)	1
**CD3+CD4+abs**	**580 (410-755)**	**1190 (724-2100)**	**0.012**	**502 (356-638)**	**1040 (904-2050)**	**<0.001**
**CD3+CD4+CD45RA+%**	**17.5 (9-33.1)**	**8.4 (3.77-17.6)**	**0.106**	**16.8 (7.76-30)**	**9.85 (3.72-16.2)**	**<0.001**
**CD3+CD4+DR%**	**11.3 (5.8-18)**	**15.4 (8.62-29.7)**	**0.237**	**9.9 (5.15-17.2)**	**20.4 (12.1-24.1)**	**<0.001**
CD3+CD8+%	28.2 (21.8-35.2)	31.7 (30-48.1)	0.102	28.4 (20.4-35.4)	27.1 (22.8-39.5)	1
**CD3+CD8+abs**	**361 (219-556)**	**956 (541-2040)**	**0.002**	**301 (176-538)**	**890 (566-1010)**	**<0.001**
**CD3+CD8+CD57+%**	**26.2 (16.5-42.1)**	**49.1 (34.7-62.3)**	**0.039**	**30.7 (17.1-47.2)**	**46.1 (39.9-64.7)**	**<0.001**
**CD3+CD8+CD57abs**	**82.8 (38-191)**	**306 (175-1440)**	**0.003**	**87.8 (42.1-180)**	**355 (242-573)**	**<0.001**
CD3+CD8+DR%	22.1 (12.3-32.4)	19.4 (11.8-30.9)	0.840	18.9 (10.8-31.2)	14.4 (13.5-36.6)	1
CD3+CD8+EE%	9.6 (6.3-14.8)	13.4 (9.3-17.8)	0.161	8.6 (5.2-14.9)	8.75 (7.15-14.2)	1
CD3+CD8+LE%	25 (10.6-51)	25.4 (19.3-66.7)	0.441	23 (11-51.7)	39.3 (18.3-60.8)	0.400

Mann-Whitney is used to compute the comparison. Parameters with significant associations are presented in bold. The lymphocytic populations are expressed as median and interquarile range. Abbreviations: % percentage; abs: absolute count; DR+: HLA-DR+; EE: early effector (CD28-CD27+); LE: late effector (CD28-CD27-).

Supplementary table 12. Comparison of different T cell populations in patients with or without CMV infection

Population	Timepoint1			Timepoint 2		
	**No**	**Yes**	**p**	**No**	**Yes**	**p**
CD3+%	79.1 (72.2-85.5)	84.2 (74.7-87.3)	0.229	77.5 (67.4-84.6)	85.6 (73.5-88.5)	0.097
CD3+abs	897 (684-1350)	1260 (922-1620)	0.057	870 (556-1100)	806 (614-1420)	0.793
CD3+CD4+	44.9 (36.3-54.8)	35.2 (32.8-50.6)	0.149	44.6 (35.7-54.8)	39 (25.6-49.9)	0.185
CD3+CD4+abs	541 (386-770)	547 (451-772)	0.507	514 (338-643)	455 (342-576)	0.638
CD3+CD4+CD45RA+%	16.8 (7.25-32.9)	16.7 (5.95-22.8)	0.438	16.5 (7.22-25.9)	9.6 (4.55-16.5)	0.103
CD3+CD4+DR%	12 (6.22-18.6)	19.1 (10.8-24.8)	0.146	10.6 (5.15-20.2)	13.4 (9.1-20.7)	0.190
CD3+CD8+%	28.2 (21.5-32.8)	31.7 (25.3-45.2)	0.080	25.1 (19.9-32.6)	34.9 (22.4-50.4)	0.058
CD3+CD8+abs	342 (166-554)	415 (342-932)	0.059	271 (148-492)	287 (256-896)	0.231
**CD3+CD8+CD57+%**	**28.2 (16.7-44.9)**	**41.8 (37.6-48.7)**	**0.023**	**30.5 (16.4-46.9)**	**51.7 (43.2-57.9)**	**0.001**

Population	Timepoint1<br>No	 Yes	 p	Timepoint 2<br>No	 Yes	 p
**CD3+CD8+CD57abs**	**81.2 (36-168)**	**196 (134-442)**	**0.007**	**77.4 (31.7-192)**	**175 (111-382)**	**0.010**
**CD3+CD8+DR%**	**21.7 (13.3-31.6)**	**34.3 (22.8-45.8)**	**0.038**	**17.9 (11.4-31.2)**	**31.4 (21.2-38.6)**	**0.042**
CD3+CD8+EE%	10.6 (7.28-16.3)	9.6 (6.8-12.1)	0.646	9.55 (5.7-15.6)	6.9 (3.4-13.6)	0.199
**CD3+CD8+LE%**	**21.2 (10.5-47.1)**	**50.5 (47.6-54.6)**	**0.003**	**21.9 (9.85-46.4)**	**57 (48.6-69.9)**	**<0.001**

CMV data are available in 91 patients. Mann-Whitney is used to compute the comparison. Parameters with significant associations are presented in bold. The lymphocytic populations are expressed as median and interquarile range. Abbreviations: % percentage; abs: absolute count; DR+: HLA-DR+; EE: early effector (CD28-CD27+); LE: late effector (CD28-CD27-).
