# Supplementary material for: Expansion of CD57+ CD8 T cells in common variable immunodeficiency with hepatopathy and CMV infection
Source: Front Immunol. 2025 May 27;16:1577934. doi: 10.3389/fimmu.2025.1577934 (PMC12149213; doi:10.3389/fimmu.2025.1577934)
Supplement: Supplementary file 2 [file Image1.pdf]

EXPANSION OF CD57+ CD8 T CELLS IN COMMON VARIABLE IMMUNODEFICIENCY (CVID) WITH HEPATOPATHY AND CMV INFECTION

Patrick Bez†[1,2,3], Enrico Santangeli†[1,4], Sigune Goldacher[1,2], Ulrich Salzer[1,2], Klaus Warnatz[1,2*]

1. Division of Immunodeficiency, Department of Rheumatology and Clinical Immunology, Medical Center - University of Freiburg, Faculty of Medicine, University of Freiburg, Freiburg, Germany;
2. Center for Chronic Immunodeficiency, Medical Center - University of Freiburg, Faculty of Medicine, University of Freiburg, Germany
3. Rare Diseases Referral Center, Internal Medicine I, Ca' Foncello Hospital, AULSS2 Marca Trevigiana, Treviso
4. Department of Pediatrics, ASST Spedali Civili of Brescia, Department of Clinical and Experimental Sciencies, University of Brescia, Brescia, Italy

† These authors contributed equally to this work and share first authorship.

SUPPLEMENTARY FIGURES

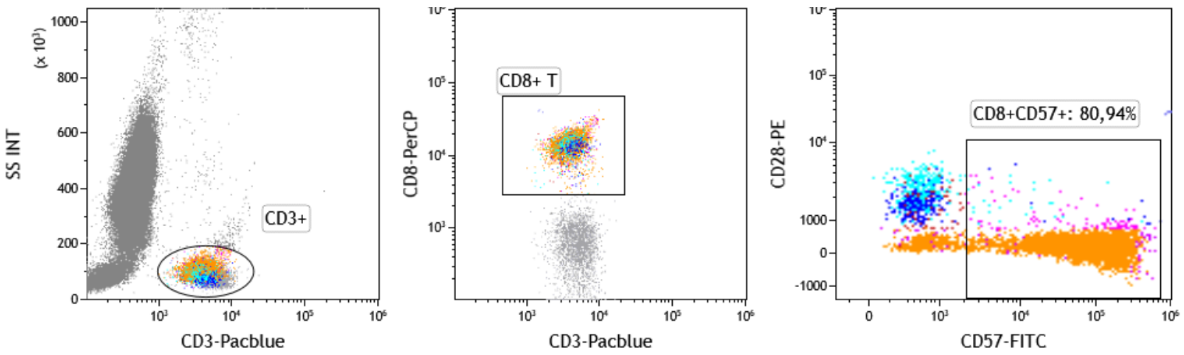

Supplementary Figure 1. Gating strategy for CD57+ CD8 T cells. Cells were first gated for CD3+ versus SSC, then for CD8+ cells versus CD3+ cells and for the quantification of CD8+CD57+ cells of total CD8+ cells a rectangular gate was set in a CD28+ versus CD57+ plot.

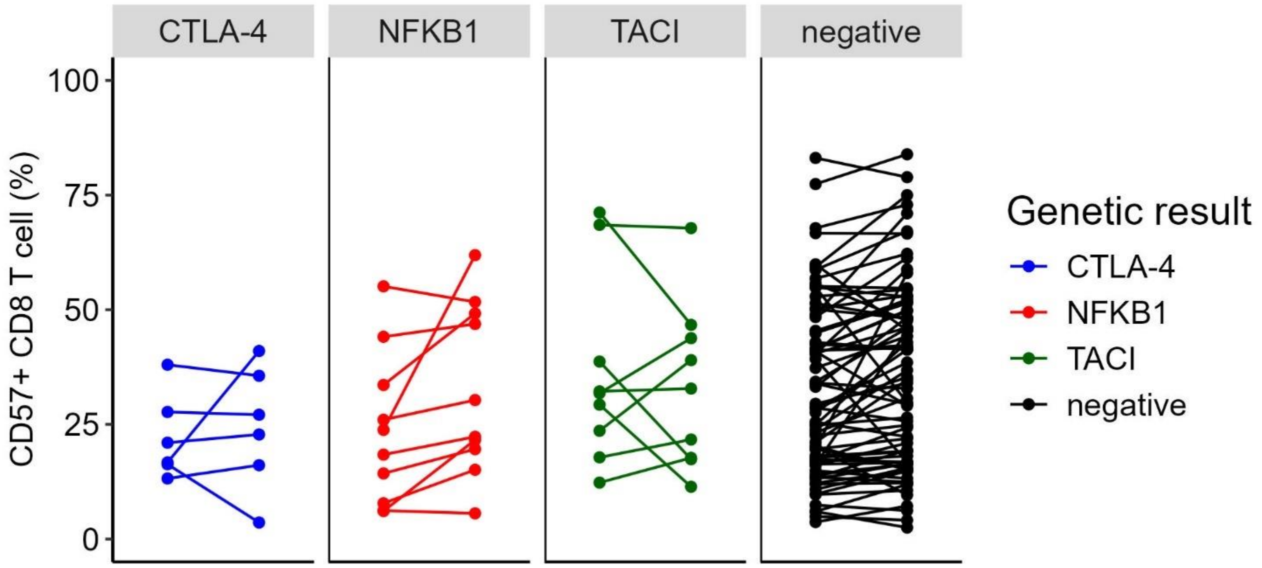

Supplementary Figure 2. Percentage of CD57+ CD8 T cell percentage in patients with defined genetic background. The figure depicts the relative counts of CD57+ CD8 T cell of the two-time points for the three most common monogenetic findings. At both time points, the Kruskal-Wallis test showed no difference between the 4 groups.

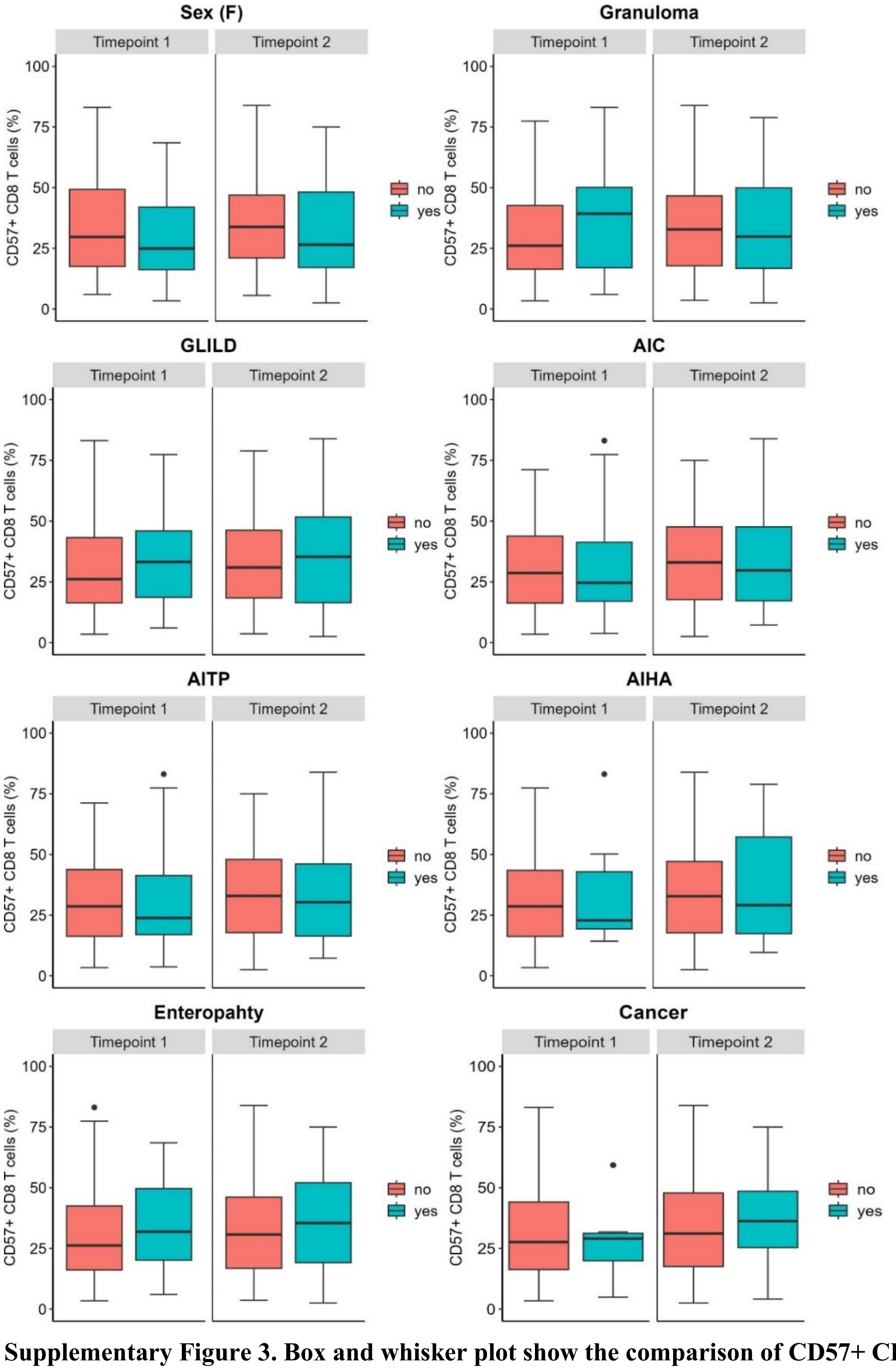

Supplementary Figure 3. Box and whisker plot show the comparison of CD57+ CD8 T cell percentages by clinical complications. The box plot represents median and the interquartile range; the vertical line the range of 5th and 95th percentile; the dots represent outliers (values above or below 5th and 95th percentile, respectively). Mann-Whitney U test was used to compare the distribution of CD57+ CD8 T cell. All the p-values did not reach the threshold of significancy. Abbreviations: GLILD= granulomatous/lymphocytic interstial lung disease; AIC= Autoimmune Cytopenia; AITP= Autoimmune Thrombocytopenia, AIHA= Autoimmune Haemolytic Anemia.

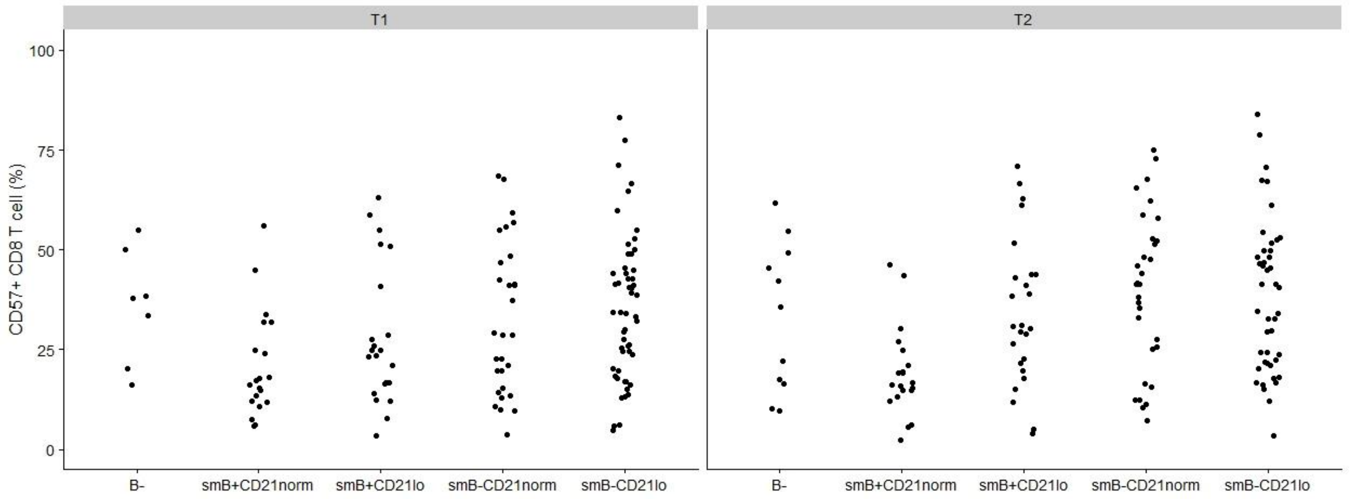

Supplementary Figure 4. CD57+ CD8 T cells grouped according to EUROClass classification.[4]
B- indicates patients with a percentage of B cells <1% of total lymphocytes; smB- is considered when switched memory B cells were less or equal to 2% of total B cells; CD21lo is considered when CD21lowCD19high B cells were higher than 10% of total B cells.

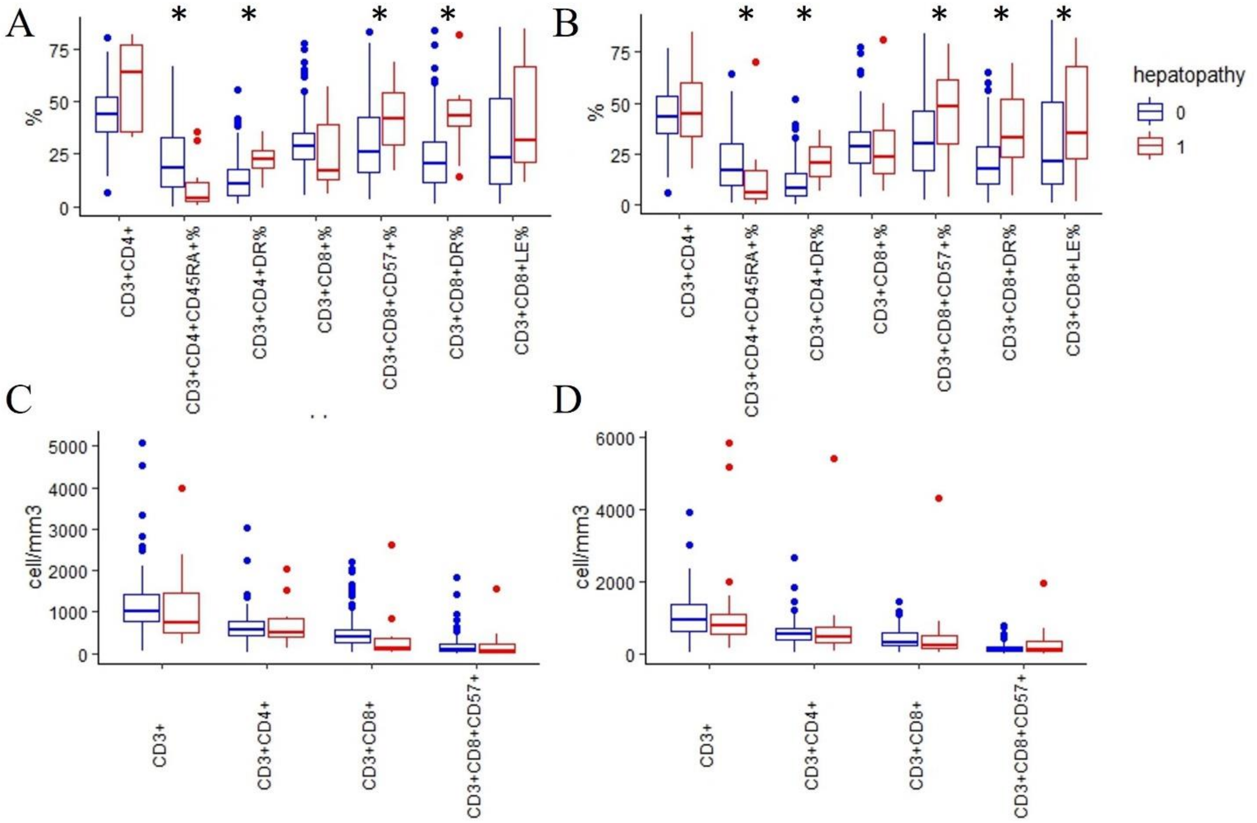

Supplementary Figure 5. Comparison of different T cell populations in patients with and without hepatopathy. A and B relative T cell counts at first and second time point, respectively; C and D absolute values at first and second phenotype. The box plot represents median and the interquartile range; the vertical line the range of 5th and 95th percentile; the dots represent outliers (values above or below 5th and 95th percentile, respectively); Mann-Whitney U test was used to compare the distribution of the different populations between groups. * represents a p-value <0.05. Abbreviations: LE: late effector.

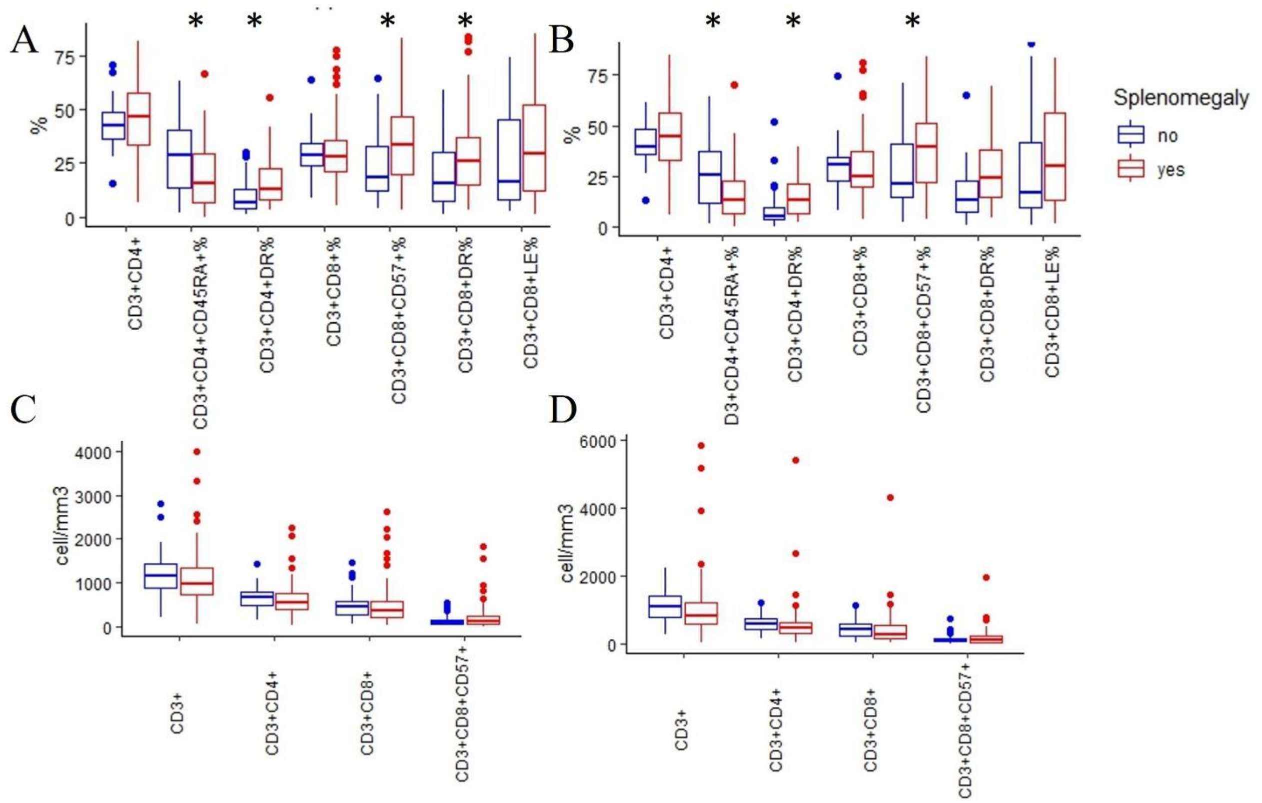

Supplementary Figure 6. Comparison of different T cell populations in patients with and without splenomegaly. A and B relative T cell counts at first and second time point, respectively; C and D absolute values at first and second phenotype. The box plot represents median and the interquartile range; the vertical line the range of 5th and 95th percentile; the dots represent outliers (values above or below 5th and 95th percentile, respectively). Mann-Whitney U test was used to compare the distribution of the different populations between groups. * represents a p-value <0.05. Abbreviations: LE= late effector.

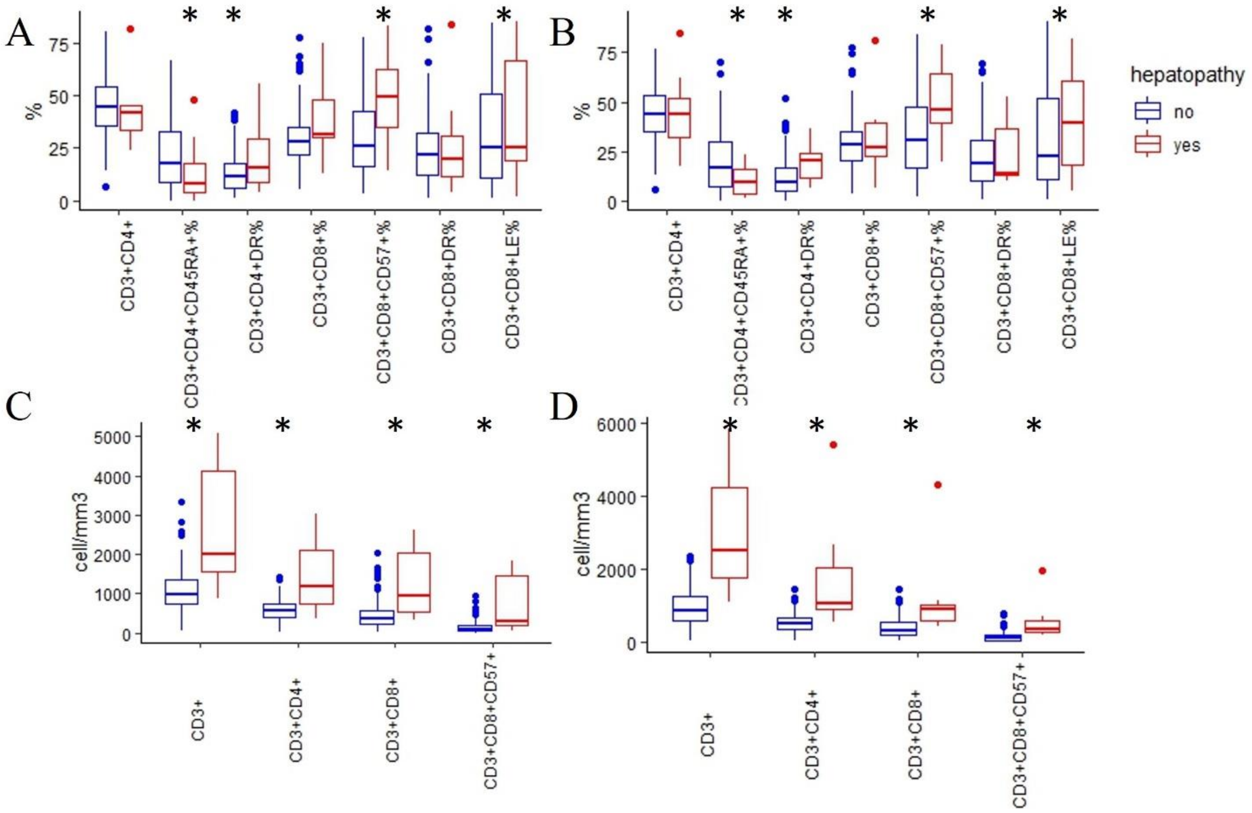

Supplementary Figure 7. Comparison of different T cell populations in patients with and without splenectomy. A and B relative T cell counts at first and second time point, respectively; C and D absolute values at first and second phenotype. The box plot represents median and the interquartile range; the vertical line the range of 5th and 95th percentile; the dots represent outliers (values above or below 5th and 95th percentile, respectively). Mann-Whitney U test was used to compare the distribution of the different populations between groups. * represents a p-value <0.05. Abbreviations: LE= late effector.

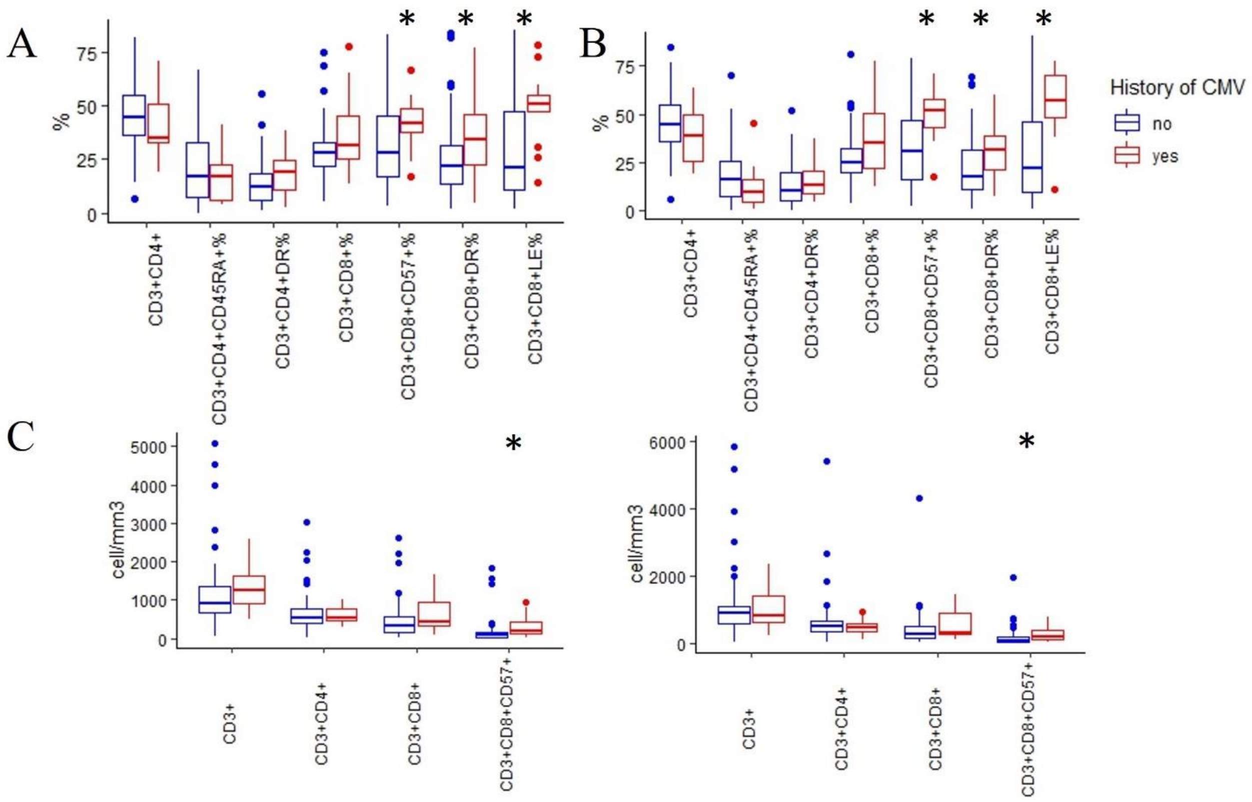

Supplementary Figure 8. Comparison of different T cell populations in patients with and without history of CMV infection. A and B relative T cell counts at first and second time point, respectively; C and D absolute values at first and second phenotype. The box plot represents median and the interquartile range; the vertical line the range of 5th and 95th percentile; the dots represent outliers (values above or below 5th and 95th percentile, respectively). Mann-Whitney U test was used to compare the distribution of the different populations between groups. * represents a p-value <0.05. Abbreviations: LE= late effector.
